# *E. coli* metabolic protein aldehyde-alcohol dehydrogenase-E binds to the ribosome: a unique moonlighting action revealed

**DOI:** 10.1038/srep19936

**Published:** 2016-01-29

**Authors:** Manidip Shasmal, Sandip Dey, Tanvir R. Shaikh, Sayan Bhakta, Jayati Sengupta

**Affiliations:** 1 Structural Biology & Bio-Informatics Division, CSIR-Indian Institute of Chemical Biology, 4, Raja S.C. Mullick Road, Kolkata-700 032, India; 2 Structural Biology Programme, Central European Institute of Technology, Masaryk University, Brno, Czech Republic

## Abstract

It is becoming increasingly evident that a high degree of regulation is involved in the protein synthesis machinery entailing more interacting regulatory factors. A multitude of proteins have been identified recently which show regulatory function upon binding to the ribosome. Here, we identify tight association of a metabolic protein aldehyde-alcohol dehydrogenase E (AdhE) with the *E. coli* 70S ribosome isolated from cell extract under low salt wash conditions. Cryo-EM reconstruction of the ribosome sample allows us to localize its position on the head of the small subunit, near the mRNA entrance. Our study demonstrates substantial RNA unwinding activity of AdhE which can account for the ability of ribosome to translate through downstream of at least certain mRNA helices. Thus far, in *E. coli*, no ribosome-associated factor has been identified that shows downstream mRNA helicase activity. Additionally, the cryo-EM map reveals interaction of another extracellular protein, outer membrane protein C (OmpC), with the ribosome at the peripheral solvent side of the 50S subunit. Our result also provides important insight into plausible functional role of OmpC upon ribosome binding. Visualization of the ribosome purified directly from the cell lysate unveils for the first time interactions of additional regulatory proteins with the ribosome.

The translation of the message encoded in the messenger RNA into protein is performed by the ribosome, a large ribonucleoprotein assembly, together with its accessory factors^1^,^2^,^3^. A number of ribosomal protein factors that are involved in the four major steps during protein synthesis (namely: initiation, elongation, peptide release, and recycling) have been structurally and functionally well characterized^1^. However, there is growing evidence that, beyond the canonical translation factors, a plethora of additional non-ribosomal factors interact with the protein synthesis machinery particularly during the ribosome assembly process, under native intracellular conditions^4^,^5^,^6^,^7^.

Many of the non-ribosomal factors are bound to ribosome in a salt-dependent manner^8^,^9^,^10^. Structural studies on the ribosome are mostly done on *in vitro* reconstituted complexes of the ribosome that are purified with a high salt wash in order to remove factors other than ribosomal proteins. We therefore envisaged that, by modulating salt conditions during purification, ribosomes isolated from cell extract might provide crucial information on association of as-yet uncharacterized factors with the ribosome. To this end, we have purified *E. coli* ribosome under slightly low-salt wash conditions. Strong association of two proteins, aldehyde-alcohol dehydrogenase E and outer membrane protein C (AdhE and OmpC, respectively), with the 70S ribosome is identified by sodium dodecyl sulfate–polyacrylamide gel electrophoresis (SDS–PAGE), followed by mass spectrometry analysis suggesting moonlighting activity^11^ of these proteins. Here, we illustrate direct interactions of these two proteins with the *E. coli* 70S ribosome.

*Escherichia coli* AdhE (~96 KDa), most likely the evolutionary product of a gene fusion, is a multitasking protein. Under anaerobic conditions, this enzyme harbours activities of three distinct proteins: alcohol dehydrogenase, acetaldehyde-CoA dehydrogenase, and pyruvate formate-lyase (PFL) deactivase^12^,^13^,^14^. However, this multifunctional protein is also synthesized under aerobic conditions, albeit at a reduced amount, but its function in the presence of oxygen largely remains to be elucidated^14^,^15^. OmpC (~40 KDa), a cation-selective porin in *E. coli*, is known to form trans-membrane pores spanning the outer membrane^16^,^17^.

Until now, stable association of these proteins with the ribosome has remained unknown. However, recent proteomic studies have indicated interaction of AdhE with the ribosome^4^,^18^. An indirect relationship between OmpC and the translation apparatus has been implicated in limited studies also where it was shown that OmpC deletion reduces cell viability, which is strongly correlated with the level of ribosomes in cell^19^,^20^. Furthermore, co-expression of the *ompC* gene with the gene of an rRNA modification enzyme has been identified in recent protein interaction network studies^4^,^6^,^21^.

A cryo-EM 3D reconstruction of the low salt washed 70S ribosome allows us to localize defined densities corresponding to the protein masses: AdhE on the head of the 30S subunit adjacent to the mRNA entrance, and OmpC on the solvent side of the 50S subunit.

Intriguingly, our study also demonstrates RNA helix-unwinding ability of AdhE. Our finding led us to propose that protein AdhE is likely involved in unwinding of specific, structured mRNAs at the 3′ end, at least under certain conditions. Although it was evidenced by Noller’s group that ribosomal proteins S3 and S4 possess weak mRNA helicase activity^22^,^23^, so far, in bacteria, no ribosome-interacting factor has been identified that shows mRNA helicase activity upon binding at the mRNA entry path. Thus, AdhE may represent a missing piece of information in the study of the unwinding process of downstream mRNA helices. The growth conditions we have used may result in cultures that are partly anaerobic (intermediate expression level of AdhE under these conditions is expected). Thus, we speculate that AdhE might exert its helicase activity in specific cellular environments.

## Results and discussion

### Ribosome-associated additional proteins

In this report we set out to investigate hitherto unknown association of non-ribosomal proteins with the ribosome. To this end, we followed a relatively unconventional approach. Traditionally, for structural studies, ribosome complexes are made by *in vitro* reconstitution methods where ribosomes purified under high salt wash condition are incubated with excess protein factors. Here, purification of ribosomes from *E. coli* under low salt wash condition was performed. We propound this mimics more physiological condition compared to the conventional *in vitro* reconstitution approach of studying ligand interaction with ribosome.

**Figure 1:**
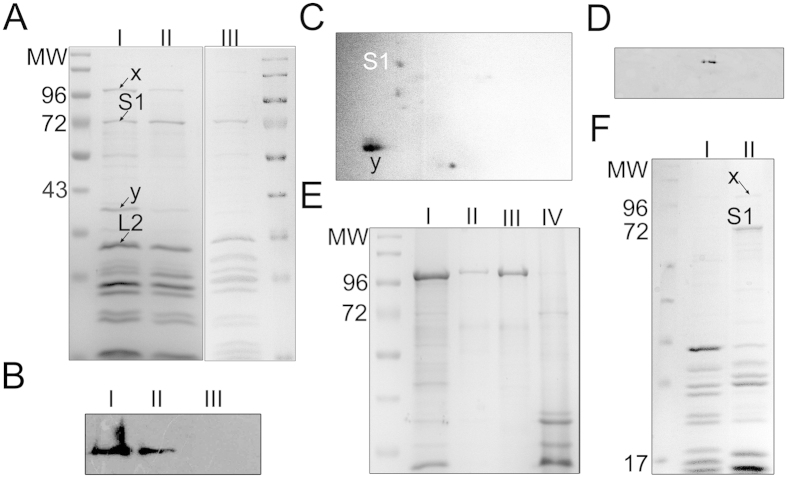
Association of additional proteins with *E. coli 70S*_*lw*_ ribosome. (**A**) 12% SDS-PAGE gel of *E. coli* 70S_lw_ (lane I following protein markers), 70S_hw_ (1M NH_4_Cl wash, lane II), 70S_hw_ (1.25M NH_4_Cl wash, lane III). Compared to 70S_hw_, strong association of two additional proteins, marked as ‘x’ and ‘y’ (ribosomal protein S1 is marked), in the 70S_lw_ ribosome is seen (identified by mass spectrometry as AdhE and OmpC respectively). (**B**) Western blot analysis of recombinant AdhE (lane I), 70S_lw_ (lane II) and 2M salt-washed70S (lane III) with anti-AdhE antibody. Presence of AdhE is clearly detected in the 70S_lw_ which is absent in high salt-washed ribosome (lane III). (**C**) 2D-PAGE protein profile of the 70S_lw_ ribosomes showing ‘y’ marked protein spot (identified by mass spectrometry as OmpC). Protein extracts from 70S_lw_ ribosomes are loaded on pH 3–10 IPG strips for IEF, followed by SDS PAGE (12% PAGE). AdhE can not be directly detected in 2D-PAGE. (**D**) Western blot analysis using anti-AdhE antibody reveals the presence of AdhE in 2D-PAGE. (**E**) Co-sedimentation assay of AdhE with 70S_hw_ ribosome is performed to validate specific binding of AdhE to the ribosome. 70S_hw_ is incubated with AdhE. After centrifugation, the pellet and supernatant are separated and resolved by 12% SDS-PAGE (lane I and II, respectively). Recombinant protein (centrifuged at the same speed) and 70S_hw_ are loaded on lane III and lane IV respectively. (**F**) Low salt-washed 70S ribosomes are split into subunits and protein contents of the 50S (lane I) and 30S (lane II) subunits are resolved by 12% SDS-PAGE gel.

The 70S ribosome pools from high- (1 and 1.25M NH_4_Cl, 70S_hw_) and low-salt wash (0.75 M NH_4_Cl, 70S_lw_) conditions were analysed by SDS-PAGE followed by MALDI-TOF mass spectrometry. It is immediately evident that, apart from the ribosomal proteins, two additional proteins are strongly associated with the ribosome in 70S_lw_ (Fig. 1A, protein bands marked as ‘x’ and ‘y’). The association of these proteins gradually decreases in higher salt washed ribosomes (Fig. 1A). Mass spectrometry analysis identified (Table S1) these two proteins as: (1) alcohol aldehyde dehydrogenase (AdhE, ~96 KDa), and (2) outer membrane protein C (OmpC, ~40 KDa). Interaction of OmpC with 70S_lw_ was further demonstrated by 2D gel electrophoresis (Fig. 1C) where intensity of AdhE appeared sparse.

Tandem MS (MSMS) analysis unequivocally identified the protein marked ‘y’ as *E. coli* OmpC (7 unique peptides for OmpC covering ~41% of the protein sequence, Table S1A). However, along with AdhE (3 unique peptides, ~5% of protein sequence covered, Table S1B) other hits were also found corresponding to the protein marked ‘x’. To further corroborate, the association of AdhE was verified by immunoblotting (Fig. 1B,D). The occupancies of the proteins were assessed from the band intensities in 1D SDS-PAGE as ~50% and ~60% for AdhE and OmpC respectively, considering the occupancy of L2 protein (binds at the intersubunit surface of the 50S subunit) as 100%.

In order to confirm that AdhE indeed has a high-affinity binding site on the ribosome, we performed a cosedimentation experiment. Purified AdhE was incubated with high salt-washed ribosomes (1.25M NH_4_Cl, 70S_hw_) and complex formation was assessed. After sucrose cushion centrifugation, the majority of the AdhE is seen in the pellet fraction in the presence of ribosomes, indicating direct interaction of AdhE with ribosomes (Fig. 1E). The AdhE protein (centrifuged at the same speed), on the other hand, is found only in the supernatant (Fig. 1E) suggesting that the event of cosedimentation of the protein with the ribosome is not just coprecipitation.

The low salt washed 70S ribosomes were split into subunits under low Mg^+2^ ions and SDS-PAGE profile of the purified 30S and 50S subunits shows the presence of AdhE (confirmed by mass spectrometry) in the 30S subunit population (Fig. 1F).

### Binding sites of AdhE and OmpC on the ribosome

**Figure 2:**
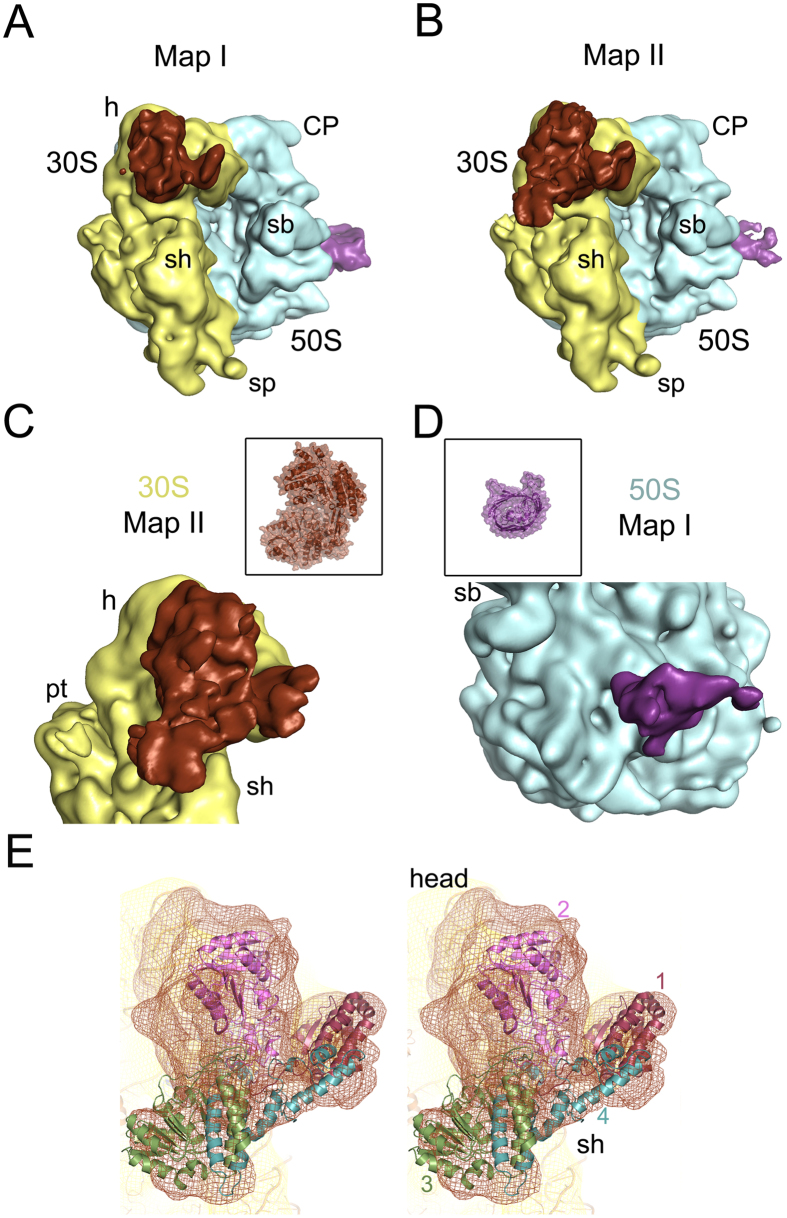
Cryo-EM reconstruction of *E. coli 70S*_*lw*_ showing AdhE and OmpC densities. 3D Cryo-EM maps, (**A**) from the entire set of particles, Map I, and (**B**) after removing 50S particles from the dataset, Map II are viewed from intersubunit side (30S subunit: yellow; 50S subunit: light blue colour). (**C**) Close-up view of the small subunit of Map II is shown with the AdhE density (brown) anchored on the solvent side of the head, close to the mRNA entrance. (**D**) The solvent side of the large subunit of Map I is viewed in close-up showing the associated OmpC density (purple). Thumbnail views of atomic structures of the proteins in similar orientations are inset in C and D. (**E**) Close-up view of the cryo-EM density attributed to AdhE protein (brown mesh) with the model structure (domain-wise coloured ribbons) is shown in stereo. AdhE is anchored on the head of the 30S subunit (yellow mesh; PDB: 2I2U, orange colour) through its domains 1 and 2. Landmarks for the 30S subunit: h, head; sh, shoulder; sp, spur. Landmarks for the 50S subunit: CP, central protuberance; sb, L7/L12 stalk base.

Using cryo-EM and single particle reconstruction techniques, a density map (Map I) of low salt washed *E. coli* 70S ribosome (70S_lw_) was generated. Comparison of Map I with a previously determined map of the 70S ribosome lacking any additional protein mass immediately reveals two distinct additional masses of density on Map I (Fig. 2A, Supplemental Fig. S1C): (1) a large density at the head of the 30S subunit, closely attached to the protein cluster S3-S10-S14^24^, and the beak (helix 33 of 16S rRNA), and (2) a comparatively smaller, cylindrical density at the peripheral solvent side of the 50S subunit, adjacent to proteins L20 and L21^25^. However, this reconstruction shows low occupancy of the protein on the head of the small subunit. We noticed a subpopulation of 50S subunits that was eventually removed from the dataset by classification-based verification^26^. The resultant part of the dataset yielded Map II, which shows augmentation of the mass at the head of the small subunit (Fig. 2B, Supplemental Fig. S1D). The ligand binding sites are well correlated with the local resolution maps showing decreased resolution at the head of the 30S subunit as well as at the peripheral solvent-exposed region of the 50S subunit below the stalk base (Supplemental Fig. S2).

We have interpreted the bulkier extra mass segmented from the 30S subunit of Map II as AdhE by visual inspection (Fig. 2B,C, Supplemental Fig. S1D), considering much higher molecular weight of AdhE compared to OmpC. The smaller density at the back of the large subunit isolated from Map I (which shows higher occupancy of this protein mass) was attributed to OmpC (Fig. 2D) based on the similarity with the cylindrical shape of its reported structural core^27^. Since the 70S ribosomes were isolated directly from cell lysate, heterogeneity in sample is expected. Nevertheless, direct visualization of the protein masses on the reconstructed 3D map from the total dataset suggests that these two proteins remained bound to a significant population of the 70S ribosomes.

Although a recent *E. coli* interactome network study has implicated ribosomal protein S2 as a putative interaction partner of AdhE^4^, we did not observe any direct connection between S2 and the AdhE mass in our map. In fact, protein S2 is visible only partially. The crystal structures of the 70S ribosome^24^ showed that the protein S2 binds loosely at the hinge between the head and the body of the 30S subunit. The large globular domain of S2 is seen on the body of the 30S subunit but the coiled-coil motif made of two helices that is interacting with the head is mostly absent. Consistent with its weak and reversible binding nature^28^, no density corresponding to protein S1 is found at its known location on the platform of the 30S subunit^29^ although it is seen associated with the 70S_lw_ in SDS PAGE analysis (Fig. 1A,C), suggesting that the protein might have dissociated during grid preparation.

### Modelling reveals AdhE-ribosome interactions

No high-resolution structure is available for the full-length *E. coli* AdhE protein (891 residues). We have generated a full-length model of AdhE (details provided in Methods). According to the modelled structure, AdhE consists of four domains (Supplemental Fig. S1A). Each domain is connected to the next one with long linker region, suggesting domain movements around flexible linker regions (characterized by low-energy barriers^30^) would be allowed. Domain I, II and III show compact structures. On the other hand, domain IV consists of few structured regions, along with many unstructured loop regions. The size of the modelled AdhE structure matches well with the protein mass isolated from Map II (Fig. 2C). When the domains are separately viewed we found distinctly recognizable features in the isolated density attributed to AdhE, particularly for domains 1 and 2. The derived model is fitted domain-wise (first manually, followed by MDFF flexible fitting) reasonably well into the corresponding segmented cryo-EM density map (Fig. 2E).

Our reconstruction reveals that AdhE establishes extensive contacts with the head of the 30S subunit, interacting in two principal regions (apparently through its domains 1 and 2). An additional density (Fig. 2E and Supplemental Fig. S4B) is seen at the top of domain 2. It appears that this density corresponds to helix 41a (h41a; which remains stacked onto h41 in the crystal structures^31^) of the 16S rRNA which curls towards AdhE in our reconstruction (marked with an asterisk in Supplemental Fig. S4A). In contrast, domain 3, which extends downward, is not in contact with the ribosome. Our fitting places the C-terminal region (domain 4) of the protein in close contact with the protein S3. Densities corresponding to domains 1 and 2 of AdhE are very strong yielding a cross correlation value coefficient of 0.8. In contrast, scattered density at higher contour level corresponding to domain 3 suggests conformational variability of this domain (cross correlation value of 0.7). It is clear that domain 1 and 2, due to strong interactions with the ribosome, are stably bound, while domain 3 has substantial degrees of freedom.

**Figure 3:**
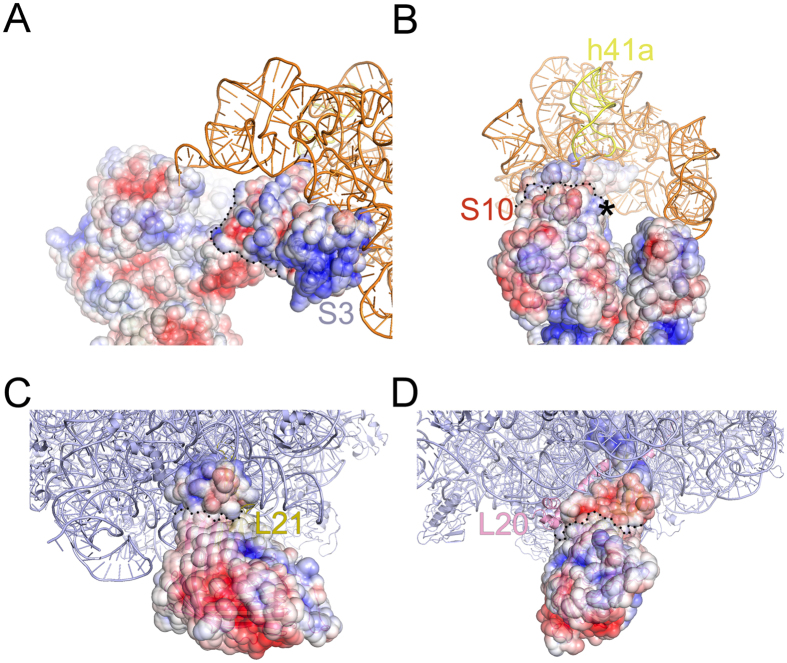
Surface potential of AdhE and OmpC with their protein neighbours. Surface potentials (blue, positive; red, negative potentials), indicating complementary charge distribution of AdhE (domains 1 and 2) with (**A**) S3 and (**B**) S10 are shown with coordinates of 16S rRNA head domain (orange). The possible interacting site of h41a with domain 2 of AdhE is marked with an asterisk in (**B**). Surface potentials of OmpC and interacting protein neighbours (**C**) L20 and (**D**) L21 are shown with the 50S subunit coordinate (PDB: 2I2V, slate colour). The distribution of the charges indicates juxtaposition of opposite polarity in both the cases. The boundaries for both AdhE and OmpC on overlapping surfaces are marked with dotted lines in each panel.

The salt sensitivity of the two proteins indicated that complementary charge interactions are likely responsible, at least partially, for their association with the ribosome. Qualitative examination of the electrostatic potential at the interface of AdhE: S3 and AdhE: S10 shows that the regions of AdhE facing S3 and S10 are of opposite polarity, lending to apparent stabilization (Fig. 3A,B). The antipolar juxtaposition of electrostatic surface potentials of the proteins further supports the localization of the atomic model of ribosome-bound configuration of AdhE. We refrain from further analysis of the protein-ribosome interactions in absence of the high resolution structure of the AdhE protein.

### Implications for the functional role of AdhE

AdhE is anchored on the head of the 30S subunit and contacts a large area of the peripheral solvent-exposed surface with three of its domains (1, 2 and 4), bridging the groove between protein S3 and the beak region. The mass corresponding to domain 3 extends into the entrance of the mRNA channel.

**Figure 4:**
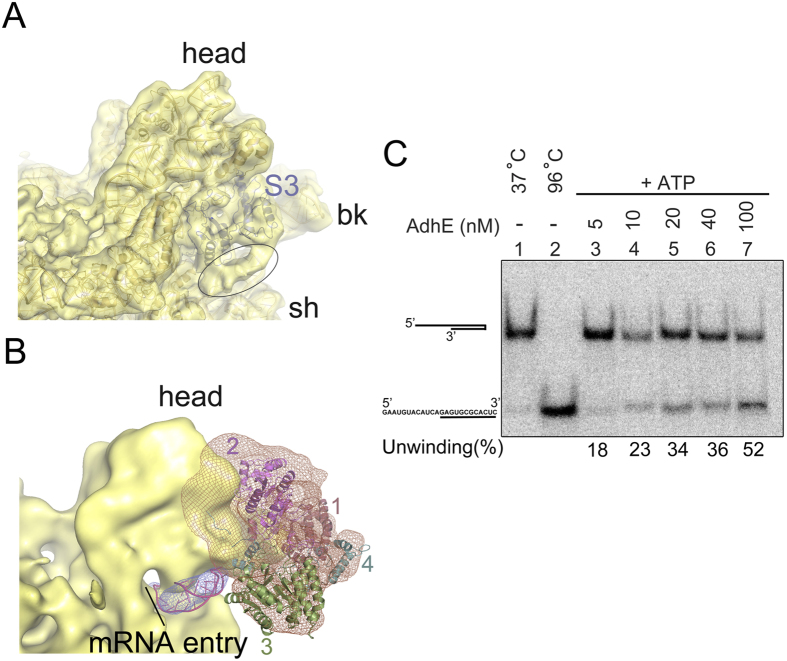
Visualization of mRNA bundle at the channel entrance and RNA duplex unwinding activity of AdhE. (**A**) Extra density identified as an ‘mRNA bundle’ at the mRNA entrance, adjacent to S3, is encircled in the cryo-EM map EMD-5036. (**B**) The AdhE-bound 30S subunit of Map II is viewed along with the extra density segmented out from EMD-5036 showing its accessibility to domain 3 of AdhE. A fragment of double helical RNA (purple, 25 nucleotides) can be nicely accommodated into the density (blue mesh) attributed to ‘mRNA bundle’. (**C**) Gel retardation assay of AdhE and duplex RNA substrate in presence of ATP (2mM). The unwinding reactions contain the radiolabelled RNA The unwinding reactions contain the radiolabelled duplex RNA (5′-GAAUGUACAUCAGAGUGCGCACUC-3′, complementary sequence is underlined) incubated under denaturing conditions without protein (lane 1), duplex RNA denatured by heating to 96 °C (as described in ‘Methods’) (lane 2), and RNA duplex treated with different concentrations (nM) of protein AdhE (lane 3–7) in presence of ATP.

Close analysis of a previous 6.7 Å map (EMD-5036, a map of EF-Tu ternary complex bound ribosome^32^ complex containing a long synthetic mRNA^33^) shows an extra density at the mRNA entrance, which is tucked underneath protein S3. From our analysis (Fig. 4A,B) it appeared likely to be an mRNA bundle. Existence of a similar extra density at the mRNA entrance was interpreted as an mRNA bundle in a recent study also^34^. AdhE is optimally positioned to access the mRNA bundle (Fig. 4B). In addition, a recent cryo-EM study revealed similar binding region for a eukaryotic SF2 DEAH/RHA helicase family protein DHX29^35^. These observations collectively prompted us to examine the RNA helicase activity of the protein AdhE.

Since known RNA helicases usually function in ATP-dependent manner, we performed helicase assay for AdhE in presence of ATP. Our helicase assay clearly manifests that AdhE is indeed capable of unwinding structured RNA in the presence of ATP. The addition of increasing concentrations of AdhE to the assay mixture containing structured RNA and ATP resulted in increasing unwinding of RNA substrate (Fig. 4C).

AdhE has no known ATP binding site (NAD binding region residues 422–427). However, NSitePred^36^ analysis indicates domain 3 (residues 545–550) could be a potential region for nucleotide triphosphate/diphosphate binding. Alignment of an ATPase domain (PDB 2QV7) shows weak structural homology (rmsd ~3 Ǻ) with this domain (Supplemental Fig. S4C). A plausible explanation for the weak density corresponding to domain 3 of AdhE (mentioned earlier) could be that it is present in an ensemble of apo- and nucleotide binding conformations in our reconstruction.

**Figure 5:**
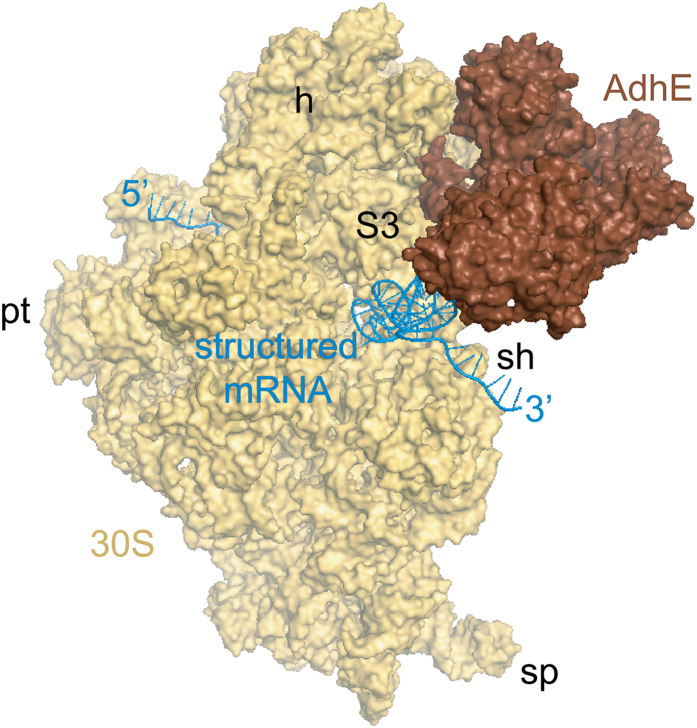
Proposed model for the ribosome-bound AdhE function. Fitted AdhE model structure (brown) is shown (surface view) along with the 30S subunit structure (pdb code: 2I2U; yellow). A hypothetical mRNA (blue) at the mRNA channel shows close proximity of the structured 3′ end to AdhE. Landmarks for the 30S subunit: h, head; sh, shoulder; pt, platform; sp, spur.

Many mRNAs possess secondary structure^37^ that needs to be disrupted for efficient translation. The previously-unknown helicase function of AdhE correlates well with its position on the ribosome and the result indicates that ribosome-bound *E. coli* AdhE may partly account for ribosome’s mRNA helicase processivity. Based on our finding, we propose that a major functional role of ribosome-associated AdhE could be to promote local unwinding of some of the mRNAs with stable stem loop structure at the 3′ end, ensuring its linear configuration at the entrance (Fig. 5). It is possible that the growth condition used in our study (glucose in rich media and growth to high density) induced partially anaerobic bacterial growth producing intermediate levels of AdhE and the protein on the ribosome helps to unwind a specific class of mRNA particularly in this cellular condition.

In the absence of high resolution structures of AdhE, the exact mechanism of mRNA helix unwinding cannot be determined. Nevertheless, the ribosomal position of AdhE would be more consistent with an active mechanism of action^38^.

Interestingly, as revealed by *in vivo* and *in vitro* ribosome assembly studies^5^,^39^, the protein cluster S3-S10-S14^24^ is assembled within the 30S ribosomal subunit at a late stage of biogenesis. Based on the extensive contacts of AdhE with S3 and S10, an additional function of AdhE at a late stage of ribosome assembly cannot be ruled out.

### Ribosome binding and function of OmpC

**Figure 6:**
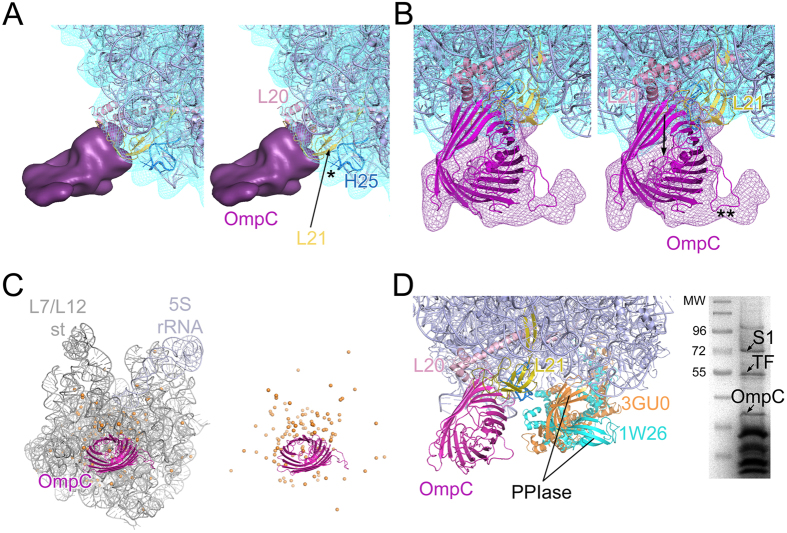
Interactions of OmpC with ribosomal neighbours and its probable functional roles. (**A**) Close-up view of OmpC (purple solid surface) in stereo showing interactions with ribosomal proteins (L20 and L21) and rRNA helices (PDB: 2I2V (slate colour) fitted inside the 50S subunit of Map I (blue mesh). One of the interaction sites shows empty density (marked with an asterisk). The closest neighbour in this region is H25 of the 23S rRNA (coloured deep blue). It appears that H25 stretches out and interacts with OmpC. (**B**) Stereo representation of OmpC density (purple mesh) with the fitted crystal structure (PDB: 2J1N; magenta colour) in close-up. The arrow indicates the hollow passage and the region of asymmetry in OmpC structure. The density corresponding to loop 4 is marked with a double asterisk. (**C**) The distribution of Mg^2+^ (orange spheres) in the 23S rRNA (adapted from PDB: 2I2V, coloured grey) is shown with the fitted coordinate of OmpC (magenta). The distribution of magnesium ions in the 23S rRNA core is very similar in all the available *E. coli* 70S crystal structures (PDBs: 2I2V, 3OFQ, 3I1N, 3I22) and is seen largely concentrated around the hollow passage, formed by the beta-barrel core structure of OmpC. (**D**) The ribosome-bound structure of trigger factor (TF) (PDB: 1W26) is aligned to the 50S subunit crystal structure (PDB: 2I2V, coloured slate) showing OmpC (magenta) binding site. When another crystal structure of trigger factor (3GU0) is aligned, it is seen that, while the N and C-terminal domains of both the crystal structures of trigger factor nicely superimpose, the PPIase domains (measures ~42 Å in length), show a large deviation. 12% SDS-PAGE gel image of *E. coli* 70S ribosome purified under 0.5 M NH_4_Cl salt-wash condition shows association of TF (identified by mass spectrometry) with the ribosome.

Apart from the large mass of AdhE, another smaller density attached to the large subunit is seen in our map which has been identified as OmpC. OmpC contacts a small area (relative to the area covered by AdhE) on the external surface of the large subunit consisting of proteins L20 and L21^25^ (Fig. 6A). According to the crystal structure^27^, OmpC adopts a hollow cylindrical structure with a 16-stranded β-barrel fold (Supplemental Fig. S1B). The β-barrel core of OmpC (pdb: 2J1N) fits quite well into the mass attributed to OmpC in our reconstruction (Fig. 6B) with a cross-correlation coefficient of 0.8.

The crystal structure of OmpC^27^ revealed short connections at the peripheral rim facing the membrane, and long loops at the extracellular end. The front view of the protein mass on the 50S subunit shows a groove-like structure (marked with arrow in Fig. 6B), as in the similarly oriented view of the crystal structure (Supplemental Fig. S1B), which breaks the symmetry of the β-barrel core. Our fitting places the membrane-associated region of OmpC towards the ribosome, while the extracellular part remains solvent-exposed.

A distinct feature in the OmpC structure as suggested by the crystallographic study^27^ is a long loop at the extracellular side (Supplemental Fig. S1B). In agreement with this observation, the mass attributed to OmpC on the 50S subunit also shows a protruding density bulge (marked with double asterisks in Fig. 6B). The connection between the 50S subunit and OmpC is circumferentially sealed, and strong connections between OmpC with L20 and 21 are visible on one side (Fig. 6B). On the other side, it appears that helix 25 of the 23S rRNA, in an extended conformation in our reconstruction, interacts with OmpC (marked with an asterisk in Fig. 6A). The potential surface maps between L20:OmpC as well as L21:OmpC exhibit qualitative electrostatic complementarity (Fig. 3C,D).

The absence of data regarding a direct interaction of OmpC with the ribosome constitutes an impediment to conclusions about the functional role of the protein on the ribosome. Nevertheless, based on its position on the ribosome, it is tempting to propose probable functional roles of this protein. *E. coli* ribosomal crystal structures exhibit a patch of highly concentrated Mg^2+^ ions in the interior of the 50S subunit aligned to the OmpC hole (at the interface of domain V and domain II of 23S rRNA (Fig. 6C)). Perhaps OmpC, being a cation transporter, recruits Mg^2+^ ions inside the rRNA skeleton and consequently helps to stabilize the 50S subunit core structure. However, such a function may be required only during assembly of the 50S subunit.

Furthermore, it was reported that trigger factor (TF), a ribosome-associated chaperone, shows a strong preference for outer membrane β barrel proteins^21^,^40^,^41^. As revealed by structural studies, the ‘dragon shaped’ TF forms an arch over the tunnel exit^42^ where it contacts protein L23 *via* its N-terminal domain in the ribosome-bound state^42^,^43^,^44^. Nevertheless, the peptidyl-prolyl isomerase (PPIase) domain (~42 Å long) shows substantial rotational freedom when available TF structures are compared. This domain appears to be capable of interacting with the ribosome-bound OmpC in an altered conformation (Fig. 6D). One interpretation would be that TF binding on ribosome is stabilized by its interaction with OmpC. Interestingly, TF association along with OmpC is seen (identified by MSMS analysis) in the SDS PAGE protein profile of 70S ribosome purified under even lower (0.5 M NH_4_Cl) salt-wash condition (Fig. 6D). This observation goes in line with our above postulation.

## Conclusions

Fast kinetics of ribosome biogenesis and activity *in vivo* indicates that intra-cellular processes may be supplemented by additional factors capable of enhancing efficient assembly and protein synthesis^45^,^46^. Indeed, recent proteomic studies have indicated that numerous non-ribosomal factors in bacteria might have a crucial role in ribosome function. However, these studies fall short of providing information on specific interaction modes as well as functional roles of the additional ribosome-interacting proteins.

The research reported here unveils stable interactions of two moonlighting proteins, AdhE and OmpC, with the ribosome and 3D reconstruction using cryo-EM enabled us to localize the proteins on the ribosome. Besides, our results shed some light on their plausible functional roles upon ribosome binding. Thus, this study paves the way for future research aimed at understanding the involvement of two as yet unknown proteins in translation regulation.

## Methods

### Purification of the *E. coli* 70S ribosome under different salt-wash conditions

70S ribosomes were isolated from log phase growth (OD_600_ 0.65) of *E. coli* MRE600 cells and purified as described previously^47^,^48^,^49^ under low salt conditions. The concentration of NH_4_Cl was carefully maintained each time during the salt wash step. The remaining steps were the same as described in Das *et al.*^47^. The 70S ribosomes (low salt washed) were further centrifuged through a 10–40% sucrose gradient without Mg^+2^ to isolate the 30S and 50S subunits.

### Gel electrophoresis and western blot analysis

Ribosome samples with or without bound AdhE protein (70S_lw_ and 70S_hw_ respectively) were loaded on a 12% denaturing SDS-PAGE gel and stained with Coomassie blue for visualizing protein bands. AdhE was localized and estimated to have molecular weight of ~97 KDa.

We used 0.6M NH_4_Cl washed 70S ribosome to extract ribosomal protein (for 2D PAGE). 67% acetic acid solution with 30 mM Mg^+2^ (extraction buffer) was used. Precipitated RNA was separated by centrifugation. The pellet was washed several times with extraction buffer to extract maximum amount of protein. In order to remove acetic acid, supernatant was dialyzed overnight at 4 °C and concentrated to 150 μl. Methanol (4 times) was added to the concentrated supernatant and mixed well. Then 150 μl of chloroform and 450 μl water (HPLC grade) were added and mixed thoroughly. The mixture was centrifuged at 5585g for 10 min. Pellet formed between upper and lower phases and the upper phase was discarded. 450 μl methanol was added and thoroughly mixed. Following centrifugation at 5585g for 15 min., protein pellet was dried for 10 min at room temperature. Rehydration buffer (8M urea, 4% 3-[(3-cholamidopropyl) dimethylammonio]-1-propanesulfonate (CHAPS)), 2M thiourea and 0.001% bromophenol blue) was added to make the final volume to 125 μl and the mixture was kept at 4 °C, overnight with slow agitation. For isoelectric focusing we used an IPG strip (BioRad), linear pH gradient from 3–10. 125 μl rehydrating mixture was added to the IPG strip and it was covered with mineral oil to prevent dehydration and was actively rehydrated at 50V for 12 hr. The isoelectric focusing was carried out according to BioRad protocol. IPG strip equilibration was done using equilibration buffer 1 (6M urea, 20% SDS, 0.05M Tris-HCl pH-8.8, 20% glycerol and 2% DTT) for 30 min, followed by equilibration buffer 2 (where 2% DTT was substituted by 2.5% IAA) for another 30 min. The IPG strip was then loaded onto 12% SDS-PAGE gel. ImageJ (http://imagej.nih.gov/ij/) was used to quantify the band intensities of the 1D SDS-PAGE gel.

Western blot analysis was performed for ribosomes with or without the bound AdhE protein (70S_lw_ and 2M salt washed 70S, respectively) and for the recombinant AdhE protein (procured from GCC Biotech, India). Blots were probed with specific antibody for AdhE (Agrisera Antibodies, Sweden). Binding of the secondary HRP-conjugated anti-rabbit antibodies (Millipore, USA) was analyzed using ImmunoCruz (Santa Cruz Biotechnology Inc., USA).

### Mass spectrometry analysis

Mass analysis was performed using 4800 MALDI TOF/TOF (model 4800, Applied Biosystems, USA) instrument operated in ‘reflectron’ mode. For MS/MS analysis protein bands were excised from the gel and digested in gel with the help of an In-Gel-Tryptic-Digestion-Kit (Thermo Pierce). Mass analysis was performed using a saturated solution of CHCA (α-cyano-4-hydroxycinnamic acid) in 50% acetonitrile/0.1% trifluoroacetic acid. The MS/MS peaks of the most intense mass ions were searched against the NCBInr Database using MASCOT software (Matrix Science Ltd., London, UK). Peptides were matched to proteins when statistically significant MASCOT probability scores (<0.05) were consistent with experimental relative molar mass (Mr) of the protein.

### Ribosome cosedimentation assay

Ribosome cosedimentation assay was performed according to Guo *et al.* with some modifications^50^. Ribosome without the bound AdhE protein were incubated with 15-fold excess of recombinant AdhE protein (GCC Biotech, India) for 30 min at 37 °C in binding buffer (20mM Tris-HCl, p.H 7.5, 50mM NH_4_Cl, 10mM Mg(CH_3_COO)_2_, 1mM DTT) and the reaction mixture was layered on top of a 34% (1 ml) sucrose cushion (binding buffer) and centrifuged at 173,000 g for 2 h 30 min at 4 °C. The supernatant and pellet were collected separately. The supernatant was dialysed, concentrated and resolved in a 12% SDS-PAGE gel alongside the pellet. As control experiments, recombinant protein (centrifuged at the same speed) and 70S_hw_ were also loaded. The presence of AdhE in the control lanes indicates that co-sedimentation of the protein occurs only due to the specific interaction of the protein with ribosome.

### Helicase assay

RNA unwinding activity of AdhE was performed according to Chandran *et al.*^51^. The substrate was derived from commercial supplier (TriLink) and was a 24nt oligonucleotide with a sequence 5′-GAAUGUACAUCAGAGUGCGCACUC-3′, in which the underlined part is self-complementary. The oligonucleotide was phosphorylated with T4 polynucleotide kinase (Fermentas) and gamma-[^32^P]-ATP, and separated from unincorporated isotopes on a Sephadex G-25 column (Roche). The specific activity of the radiolabeled oligonucleotide was measured to be ~5 × 10^5^ cpm/pmol. The labelled oligonucleotides were further purified by trizol and eluted from the gel with an elution buffer (500 mM ammonium acetate, 10 mM magnesium acetate, 1 mM EDTA, 0.1% SDS) followed by brief centrifugation. The oligonucleotides were precipitated with 2.5 volumes of 100% ethanol and the pellet was resuspended in 50 μl of hybridization buffer (20 mM Tris–HCl (pH 7.5), 500 mM NaCl, 1 mM EDTA).

RNA unwinding activity was performed *in vitro* by measuring the conversion of duplex RNA to monomers. For the assay, purified AdhE was incubated with 6 fmol (0.1 nM) of duplex RNA in a 50 μl reaction containing 10 mM Tris–HCl (pH 7.5), 5 mM MgCl_2_, 60 mM KCl, 40 mg of bovine serum albumin (BSA), 100 ng of yeast tRNA, 50 units of RNasin (Promega) and when indicated 2 mM ATP. After incubation for 30 min at 37 °C the reaction was stopped by adding 50 μl of of 20% glycerol, 0.2% SDS, 4 mM EDTA and analyzed directly by electrophoresis on a native 1×TBE–12% polyacrylamide gel (29:1). The gel was dried and visualized in a phosphorimager. ImageJ software was used for evaluating the band intensities.

### Electron microscopy and image processing

Grids for cryo-EM were prepared following standard procedures^52^. A total of 273 micrographs were recorded on a Philips FEI (Eindhoven, The Netherlands) Tecnai F20 field emission gun electron microscope operated at 200kV, equipped with low-dose kit and an Oxford cryo-transfer holder at a total magnification of 89,000Х. Data were collected using 4KX4K TVIPS CCD Camera with a physical pixel-size of 15 μm (corresponding to a pixel size of 1.69 Å on the object scale).

The image processing using SPIDER^53^ included a 3D projection alignment procedure with correction of the contrast transfer function and enhancement of the high-resolution Fourier amplitudes based on X-ray solution scattering data. Good micrographs were selected after inspection of drift, astigmatism, and the presence of Thon rings in the power spectrum, and the defocus of each micrograph is estimated on the basis of its 2D power spectrum (CTF ED operation in SPIDER) followed by grouping into 44 defocus groups with defocus values ranging from 1.6–4.6 μm. A total of 56,165 particles were selected from these micrographs following automated particle picking^54^ and visual inspection. The 3D reconstructions then followed the standard SPIDER protocols for reference-based reconstruction^55^. An *E. coli* 70S cryo-EM map (EMD-5036, after removing ligands followed by filtering to low resolution), which clearly shows the density corresponding to S1 protein, was used as the reference^32^,^56^. Multiple rounds of angular refinement with angular increments was done following which the resolution of this initial map was estimated to be 12.9 Å (FSC 0.5 cut-off; Supplemnetal Fig. S1C). A subset of remaining 50S ribosome images was removed using classification-based verification^26^, leaving 40,855 remaining particles. The resolution of the resultant map was 13.6 Å (Supplemental Fig. S1D). Local resolution maps calculated using the ResMap program^57^ shows that the resolution of core part of the maps lies within 11–12 Å.

### Modelling and fitting of atomic structures into EM maps

We used the PHYRE2^58^ (Protein Homology/analogY Recognition Engine) web servers for modelling of the AdhE protein (Swiss Prot accession number: P0A9Q7). Validation of the models is described in Supplemental Methods. The model thus obtained, was fitted (details given in Supplemental Methods) keeping each domain as rigid body into the attributable cryo-EM density isolated from Map II, followed by molecular dynamics flexible fitting (MDFF)^59^. Cross-correlation coefficients were measured for the fitted domains using SPIDER. The final fitted structure of the model was assessed using the MolProbity (http://molprobity.biochem.duke.edu/) and PDBSum (https://www.ebi.ac.uk/thornton-srv/databases/cgi-bin/pdbsum/) web servers.

The OmpC crystal structure (pdb code 2J1N) was also fitted into the ligand density isolated from Map I using MDFF keeping the beta-barrel core rigid, while the flexible loop regions on the top and bottom of the beta-barrel core were kept flexible.

### Electrostatic calculations

Electrostatic surface potential maps of the proteins, based on the Poisson Boltzmann (PB) model that constitutes the fundamental equation of electrostatics, were generated using the Adaptive Poisson Boltzmann Solver (APBS) software^60^ available with the PyMol interface (DeLano Scientific). To account for the electronic polarizability, the solute dielectric constant was set to 2 and the dielectric constant of continuum solvent to 78.5 for all PB calculations. The ionic strength of monovalent ions was set to 0.150 M with an ion-exclusion radius of 2 Å. The shape of the solute was defined by its molecular surface using a probe sphere radius of 1.4 Å. Colour code units are in ±5 kT/e.

## Additional Information

**Data availability:** The cryo-EM maps (Map I and II) have been deposited in the EMDataBank (accession codes:EMD-2970, EMD-2972).

**How to cite this article**: Shasmal, M. *et al.* *E. coli* metabolic protein aldehyde-alcohol dehydrogenase-E binds to the ribosome: a unique moonlighting action revealed. *Sci. Rep.* **6**, 19936; doi: 10.1038/srep19936 (2016).

## Supplementary Material

Supplementary Information
