# Supplementary material for: E. coli metabolic protein aldehyde-alcohol dehydrogenase-E binds to the ribosome: a unique moonlighting action revealed
Source: Sci Rep. 2016 Jan 29;6:19936. doi: 10.1038/srep19936 (PMC4731797; doi:10.1038/srep19936)
Supplement: Supplementary Information [file srep19936-s1.pdf]

Supplemental Information

Title:

***E. coli* metabolic protein aldehyde-alcohol dehydrogenase-E binds to the ribosome: a unique moonlighting action revealed**

Authors:

Manidip Shasmal[1], *Sandip Dey*[1], *Tanvir R. Shaikh*[2,] *Sayan Bhakta*[1], *Jayati Sengupta*[1,]*

Addresses:

[1]Structural Biology & Bio-Informatics Division, CSIR-Indian Institute of Chemical Biology, 4, Raja S.C. Mullick Road, Kolkata-700 032, India

[2] Structural Biology Programme, Central European Institute of Technology, Masaryk University, Brno, Czech Republic

Supplemental Methods:

Fitting of AdhE model into EM maps:

Our fitting places the C-terminal region (domain 4) of the protein in close contact with the protein S3. During MDFF, intra-domain structures of the first 3 domains (residues 1-211: domain 1; residues 218-443: domain 2; residues 458-714: domain 3) were kept rigid, while the linker regions (linkers: 212-217, 444-457, 715-721) remained flexible (along with residues 83-104, a tongue-like structure in domain 1). Consistently, all unstructured regions of domain IV (residues 810-826, 840-850) were kept flexible. The fitting of residues 722 to 864 (domain 4) into the cryo-EM density of AdhE was virtually impossible without altering the modelled structure (Supplemental Fig. S3). We fitted this region by allowing the structured parts to rotate as rigid bodies around flexible regions. The C-terminal tail (residues 865-891) is unstructured in our model making it difficult to trace its position inside the density corresponding to AdhE. However, the tail is strongly positively charged, and it is accommodated inside a negatively charged groove of protein S3 following the fitting. The three proteins S3, S10, and S14 form a tight cluster at the solvent-exposed side of the head of the 30S subunit [D.E. Brodersen et al (2002) *J Mol Biol* **316**, 725-768]. Domain 2 of AdhE interacts with the N-terminal domain of S3 and the globular domain of protein S10, while its domain 1 contacts the beak region (helix 33 (h33) of the 16S ribosomal RNA (rRNA)) (Supplemental Fig. S4A, B) where it lies in close proximity to the protein S14. Proteins S3 and S10 share a common interface with domain 2 of AdhE, indicating extensive interactions between these proteins. In fact, due to this tight interaction it is difficult to mark the interface precisely. An additional density (marked with an asterisk in Supplemental Fig S4A) is seen at the top of domain 2. It appears that this density corresponds to helix 41a (h41a; which remains stacked onto h41 in the crystal structures [Selmer, M., *et al.* (2006) *Science* **313**, 1935-1942]) of the 16S rRNA which curls towards AdhE in our reconstruction (Supplemental

Fig. S4). In contrast, domain 3, which extends downward, is not in contact with the ribosome.

Validation of the AdhE model structure:

We have compared Phyre2 model with the AdhE models (N- and C-terminals are modelled separately) available in ModBase [U. Pieper et al (2014), *Nucleic Acids Research* 42, D336-46] database. The templates used in Rosetta server [Robetta for 3D modelling; S. Raman (2009), *Proteins* 77 (suppl. 9), 89–99] for AdhE modelling are also checked. It is evident that the folds of each domain are very similar to the Phyre2 generated model although the templates are different in some cases (Supplemental Fig. S5). The top 5 models generated by Robetta server show different orientations of the domains (particularly III and IV) relative to one another (Supplemental Fig. S6). Interestingly, relative orientation of domains III and IV resembles one of the Robetta model well.

PDBsum (PROCHECK) scores for Phyre2 generated model are in acceptable range (Supplemental Table S2A). MolProbity [I.W. Davis , *et al.* (2007) *Nucleic Acids Res* **35**, W375-383] and PDBsum [R.A. Laskowski (2001), *Nucleic Acids Res.* **29**, 221-2] servers are used to validate the fitted models (Supplemental Table S2B).

Supplemental Figure Legends

Supplemental Figure S1: ***Structures of AdhE and OmpC proteins and low salt washed ribosome maps.*** (A) The Phyre2 generated model of AdhE is represented as ribbon with its domains in different colours. The inter-domain linker regions and the unstructured segments that have been rendered flexible during MDFF run are coloured yellow. (B) Crystal structure of OmpC (magenta, PDB: 2J1N) in cartoon form shows a 16 pleated beta-barrel core with shorter loops at the membrane interacting face and longer loops at the extracellular side. Loop 4, according to the crystal structure, is the hallmark feature of OmpC's extracellular

region. (C) Difference map obtained from Map I is shown along with the resolution (12.9Å; 0.5 cut off) estimated from Fourier Shell Correlation (FSC) curve. (D) The difference map obtained from Map II. The resolution of the map after removal of the 50S particles from the dataset is estimated as 13.6Å. Both the difference maps have three major density clusters: (1) on the 30S subunit head, (2) on the solvent side of 50S subunit, and (3) a density resembling tRNA at the inter subunit space.

Supplemental Figure S2: ***Local resolution estimation of the two reconstructions.*** ResMap-H2 results of Map I (A) and Map II (B) have been shown with slices through the density maps (left panel), ResMap-H2 slices (middle panel) and the range of resolutions for both maps. The results indicate decreased resolution at the sites where the ligands (*, AdhE; **, OmpC) interact in both maps. It also shows that the resolution of the core part of the maps lies within 11-12Å.

Supplemental Figure S3: ***Phyre2 generated AdhE model before and after flexible fitting.*** AdhE model generated by Phyre2 (A, brick red) and the same model after domain-wise fitting (B, green cyan) into the density attributed to AdhE are presented indicating some changes in inter-domain orientations while the intra-domain structures mostly remain unchanged following MDFF run. Domains are marked 1-4 from N-terminal to C-terminal.

Supplemental Figure S4: ***AdhE model and its interactions with the ribosome.*** (A) Stereo view shows that helix 41a (h41a, yellow) of the 16S rRNA can be fitted well in the density adjacent to domain 2 (pink) of AdhE (the coordinate of the rotated head domain is coloured orange). We suspect that h41a of the 16S rRNA, in an alternative conformation, interacts with domain 2 in this region. (B) Close-up view of the ribosome-interacting domains (1 and

2) of AdhE with the head of 30S subunit. Domain 1 of AdhE (brown) interacts with h33 of the 16S rRNA while domain 2 (pink) interacts with ribosomal proteins S3 and S10. An additional density appears at the domain 2 interacting region of AdhE density as a result of tight association of the proteins. Additionally, an empty density (marked with an asterisk in A) is seen on the top of domain 2. (C) The ATP binding domain (pink) of a kinase (diacylglycerol kinase DgkB, PDB: 2QV7) is superimposed with domain III (pale green) of AdhE (rmsd ~ 3Å). The predicted potential ADP/ATP binding site on domain III of AdhE is coloured orange which matches well with the position of the ADP (blue sticks) bound to the kinase.

Supplemental Figure S5: ***AdhE models.*** Models of AdhE domains derived from different server/database are aligned showing <2 Å RMSD. Domains are marked 1-4 from N-terminal to C-terminal.

Supplemental Figure S6: ***Models for full length AdhE generated by Rosetta.*** Top 5 Rosetta models show different orientations of the domains (particularly III and IV) relative to one another.

<u>Suppl. Table S1A</u>: MS/MS Ion Search result for 'y' marked protein

Outer membrane porin protein C(*Escherichia coli*)<br><br>gi|157161697<br><br>Nominal mass (M_r): 40398	7	NCBInr<br><br>(score: 534)	41%	**K.GETQVTDQLTGYGQWEYQIQGNTSEDNKENSWTR.V**<br><br>**K.FQDVGSFDYGR.N**<br><br>**R.NYGVVYDVTSWTDVLPEFGGDTYGSDNFMQQR.G**<br><br>**R.NTDFFGLVDGLNFAVQYQGK.N**<br><br>**K.YDANNIYLAAQYTQTYNATR.V**<br><br>**K.AQNFEAVAQYQFDFGLRPSVAYLQSK.G**<br><br>**K.INLLDDNQFTR.D**

Matched peptides shown in ***bold red*** on the protein sequence (368 residues).

1 MKVKVLSLLV PALLVAGAAN AAEVYNKDGN KLDLYGKVDG LHYFSDNKSE

51 DGDQTYVRLG FK**GETQVTDQ** **LTGYGQWEYQ** **IQGNTSEDNK** **ENSWTR**VAFA

101 GLK**FQDVGSF** **DYGRNYGVVY** **DVTSWTDVLP** **EFGGDTYGSD** **NFMQQR**GNGF

151 ATYR**NTDFFG** **LVDGLNFAVQ** **YQGK**NGSVSG EGMTNNGRGA LRQNGDGVGG

201 SITYDYEGFG IGAAVSSSKR TDAQNTAAYI GNGDRAETYT GGLK**YDANNI**

251 **YLAAQYTQTY** **NATR**VGSLGW ANK**AQNFEAV** **AQYQFDFGLR** **PSVAYLQSK**G

301 KNLGVVAGRN YDDEDILKYV DVGATYYFNK NMSTYVDYK**I** **NLLDDNQFTR**

351 DAGINTDNIV ALGLVYQF

<u>Suppl. Table S1B</u>: MS/MS Ion Search result for 'x' marked protein

Protein	No. of unique peptides	Database	Protein sequence coverage	Peptide sequences
Bifunctional acetaldehyde-CoA/alcohol dehydrogenase (*Escherichia coli*) gi|15801467<br><br>Nominal mass (M_r): 96107	3	NCBInr<br><br>(score: 208)	5%	**K.QILLDTYYGR.D**<br><br>**R.YAEIADHLGLSAPGDR.T**<br><br>**K.ILIGEVTVVDESEPFAHEK.L**

Matched peptides shown in ***bold red*** on the protein sequence (891 residues).

```
  1 MAVTNVAELN ALVERVKKAQ REYASFTQEQ VDKIFRAAAL AAADARIPLA
 51 KMAVAESGMG IVEDKVIKNH FASEYIYNAY KDEKTCGVLS EDDTFGTITI
101 AEPIGIICGI VPTTNPTSTA IFKSLISLKT RNAIIFSPHP RAKDATNKAA
151 DIVLQAAIAA GAPKDLIGWI DQPSVELSNA LMHHPDINLI LATGGPGMVK
201 AAYSSGKPAI GVGAGNTPVV IDETADIKRA VASVLMSKTF DNGVICASEQ
251 SVVVVDSVYD AVRERFATHG GYLLQGKELK AVQDVILKNG ALNAAIVGQP
301 AYKIAELAGF SVPENTKILI GEVTVVDESE PFAHEKLSPT LAMYRAKDFE
351 DAVEKAEKLV AMGGIGHTSC LYTDQDNQPA RVSYFGQKMK TARILINTPA
401 SQGGIGDLYN FKLAPSLTLG CGSWGGNSIS ENVGPKHLIN KKTVAKRAEN
451 MLWHKLPKSI YFRRGSLPIA LDEVITDGHK RALIVTDRFL FNNGYADQIT
501 SVLKAAGVET EVFFEVEADP TLSIVRKGAE LANSFKPDVI IALGGGSPMD
551 AAKIMWVMYE HPETHFEELA LRFMDIRKRI YKFPKMGVKA KMIAVTTTSG
601 TGSEVTPFAV VTDDATGQKY PLADYALTPD MAIVDANLVM DMPKSLCAFG
651 GLDAVTHAME AYVSVLASEF SDGQALQALK LLKEYLPASY HEGSKNPVAR
701 ERVHSAXTIA GIAFANAFLG VCHSMAHKLG SQFHIPHGLA NALLICNVIR
751 YNANDNPTKQ TAFSQYDRPQ ARRRYAEIAD HLGLSAPGDR TAAKIEKLLA
801 WLETLKAELG IPKSIREAGV QEADFLANVD KLSEDAFDDQ CTGANPRYPL
851 ISELKQILLD TYYGRDYVEG ETAAKKEAAP AKAEKKAKKS A
```

Suppl. Table S2A: Validation of Original AdhE model (Phyre2):

Server	Values obtained for the model	Recommended values
PDBsum	89.6% (in most favoured regions)<br><br>-0.13 (G-factor overall average score)	> 90%<br><br>> -0.5

Suppl. Table S2B: Validation of the AdhE fitted Model:

<table>
<tr><td rowspan="4">Protein Name</td><td colspan="6">SERVER</td></tr>
<tr><td colspan="2">Molprobity</td><td colspan="4">PDBsum (PROCHECK)</td></tr>
<tr><td rowspan="2">Values obtained for the model</td><td rowspan="2">Recommended values</td><td colspan="2">Values obtained for the model</td><td colspan="2">Recommended values</td></tr>
<tr><td>Residues in most favoured regions (Ramachandran plot)</td><td>G-factor</td><td>Residues in most favoured regions (Ramachandran plot)</td><td>G-factor</td></tr>
<tr><td>AdhE</td><td>83rd percentile (Acceptable)</td><td rowspan="2">100th percentile</td><td>84.5%*</td><td>-0.42</td><td rowspan="2">90%</td><td rowspan="2">>-0.5</td></tr>
<tr><td>OmpC</td><td>89th percentile (Acceptable)</td><td>75.9%**</td><td>0.02</td></tr>
</table>

* Additional allowed regions	97 aa	12.3%
Generously allowed regions	16 aa	2.0%
** Additional allowed regions	54 aa	18.4%
Generously allowed regions	13 aa	4.4%

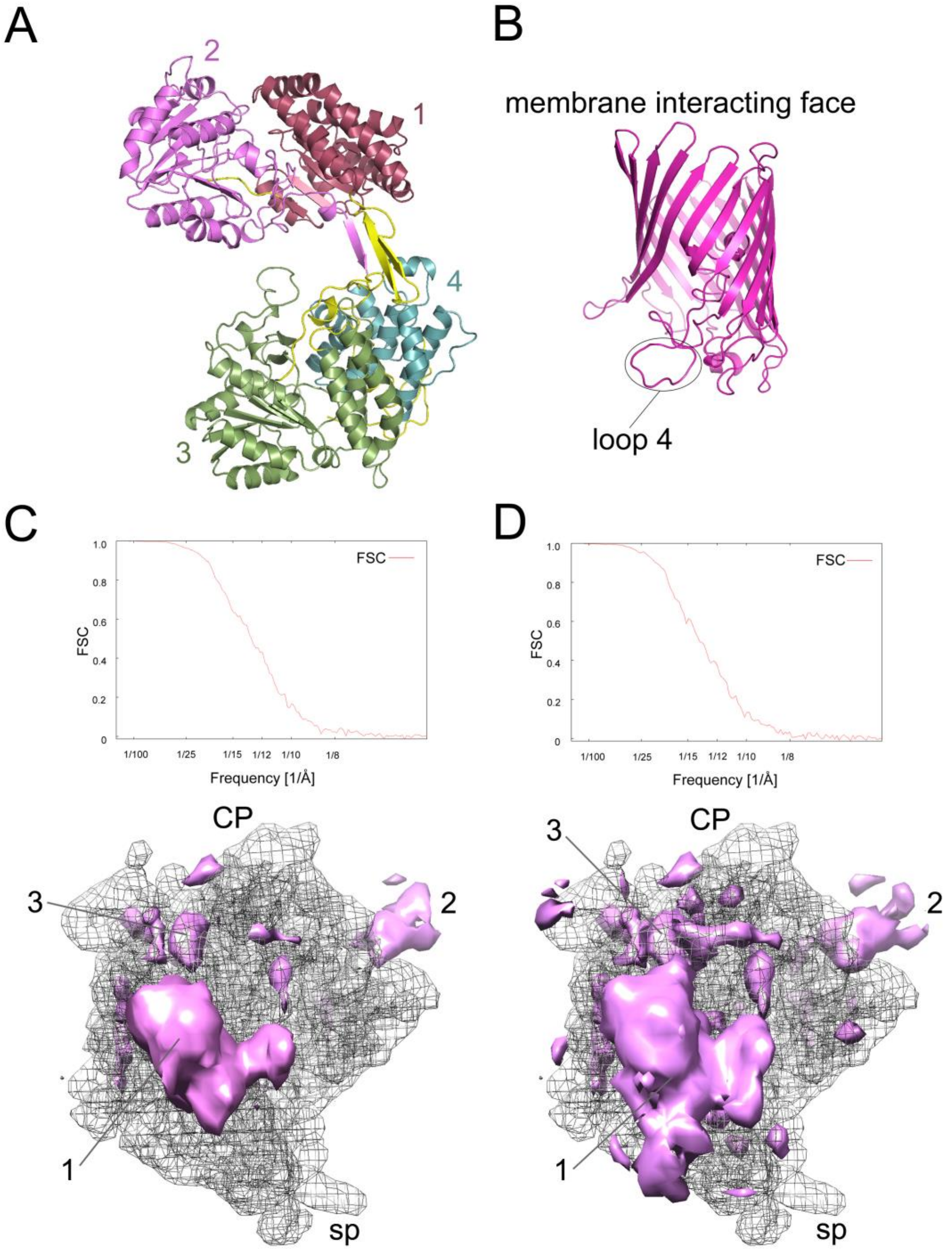
A
2
1
4
3
B
membrane interacting face
loop 4
C
D
FSC
1.0
0.8
0.6
0.4
0.2
0
1/100
1/25
1/15
1/12
1/10
1/8
Frequency [1/Å]
CP
3
2
1
sp

Fig. S1

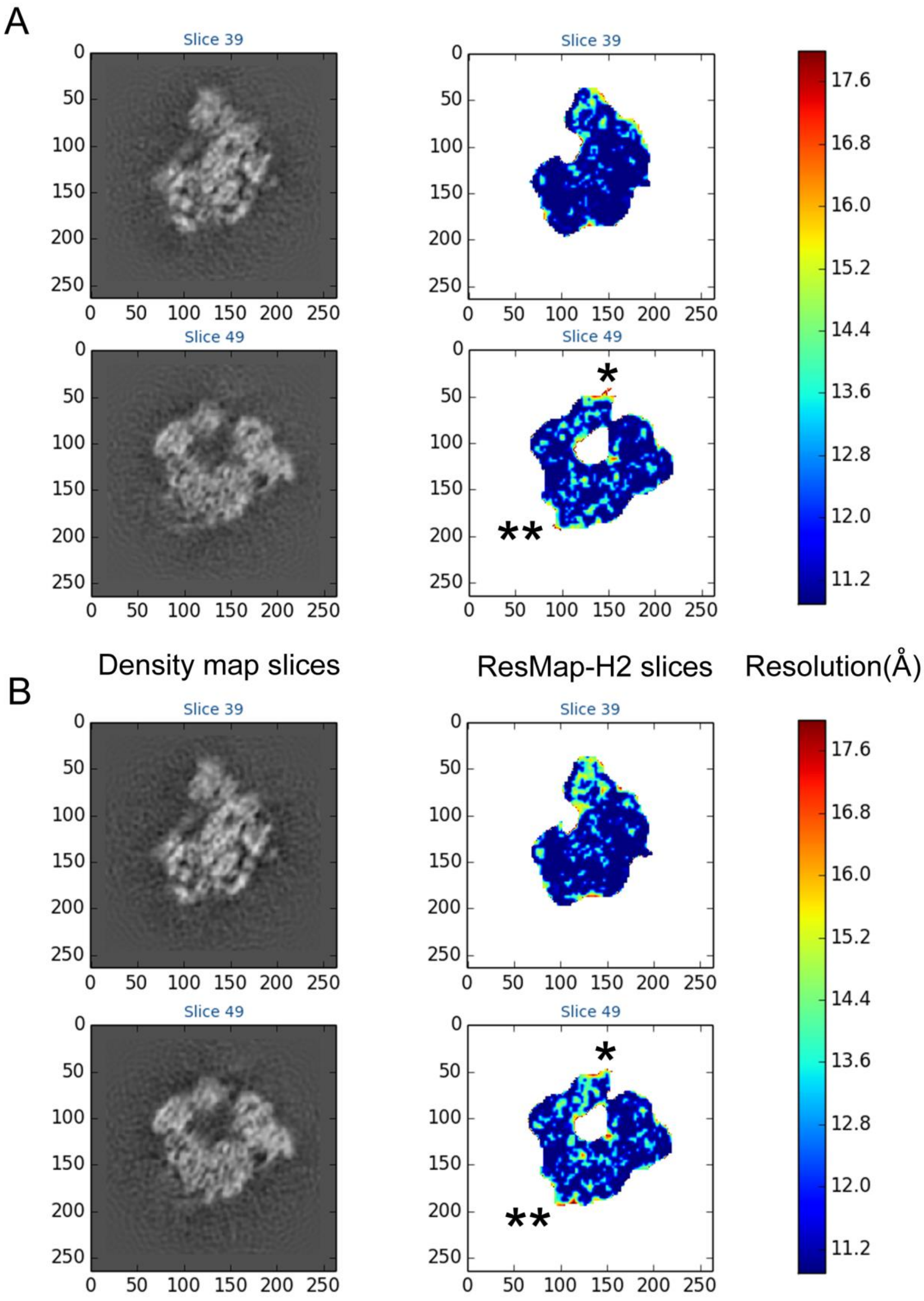
A
Slice 39
Slice 39
Slice 49
Slice 49
*
**
17.6
16.8
16.0
15.2
14.4
13.6
12.8
12.0
11.2
Density map slices
ResMap-H2 slices
Resolution(Å)
B
Slice 39
Slice 39
Slice 49
Slice 49
*
**

Fig. S2

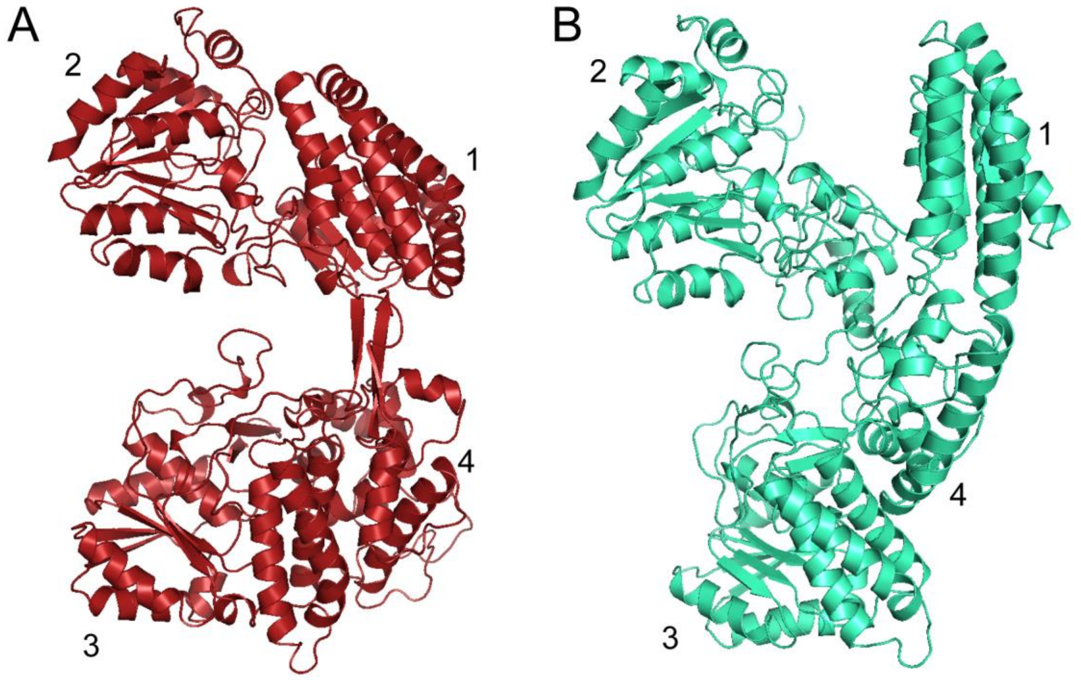

Fig. S3

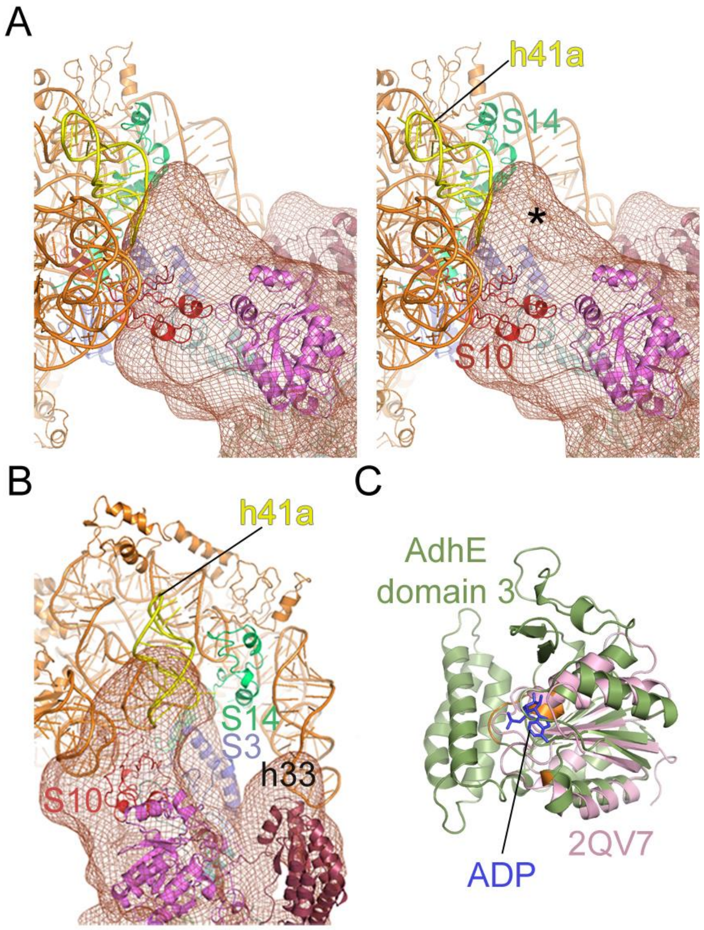

Fig. S4

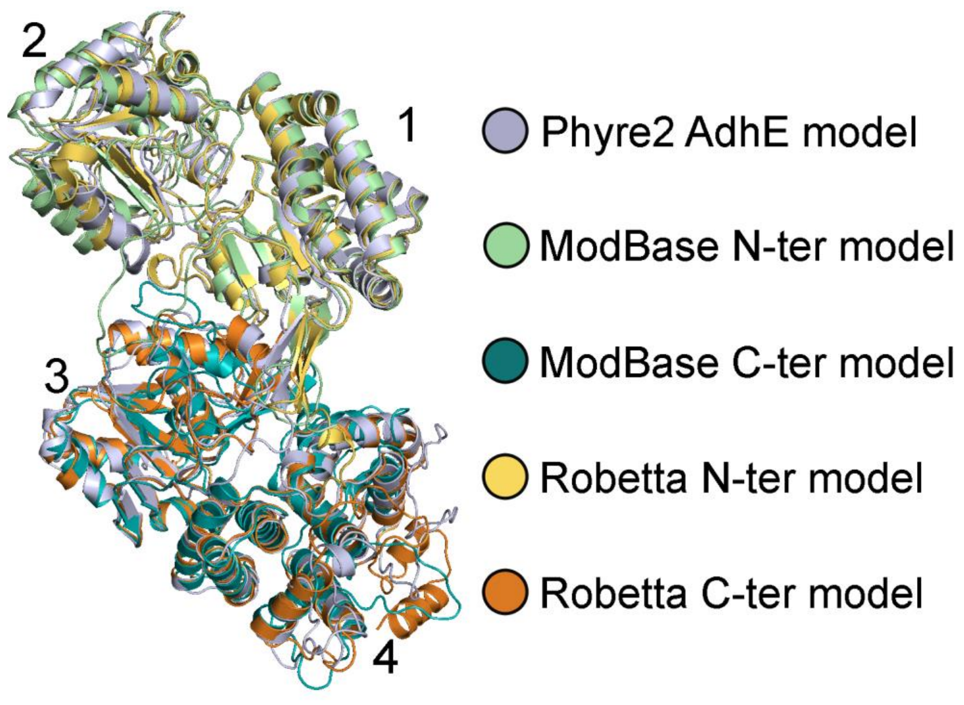
2
1
3
4
Phyre2 AdhE model
ModBase N-ter model
ModBase C-ter model
Robetta N-ter model
Robetta C-ter model

Fig. S5

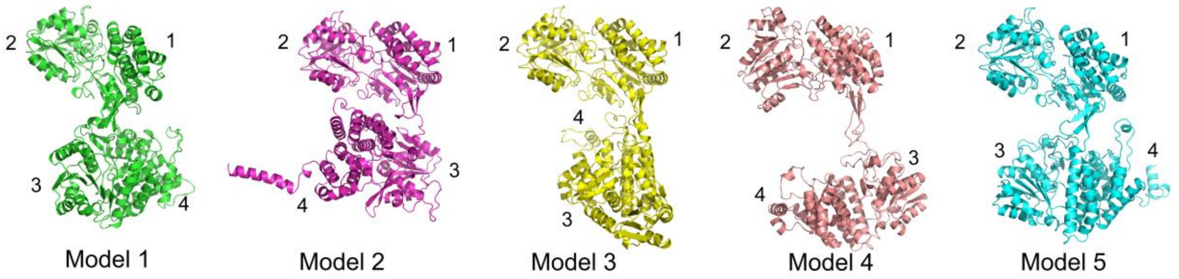
2
1
3
4
Model 1
2
1
4
3
Model 2
2
1
4
3
Model 3
2
1
3
4
Model 4
2
1
3
4
Model 5

Fig. S6
